# Frequency of cannabis use and alcohol-associated adverse effects in a representative sample of U.S. adolescents and youth (2002–2014) a cross-sectional study

**DOI:** 10.1186/s42238-020-00043-z

**Published:** 2020-10-20

**Authors:** Majed M. Ramadan, Jim E. Banta, Khaled Bahjri, Susanne B. Montgomery

**Affiliations:** 1 Department of Health Policy and Leadership, School of Public Health, University of Loma Linda, 24951 North Circle Drive, Nichol Hall 1107, Loma Linda, CA 92350 USA; 2 Center for Leadership in Health Systems, School of Public Health, Loma Linda University, 24951 North Circle Drive, Nichol Hall 1107, Loma Linda, CA 92350 USA; 3 Pharmaceutical & Administrative Sciences, School of Pharmacy, Loma Linda University, 24745 Stewart Street, Shryock Hall, Room 227, Loma Linda, CA 92350 USA; 4 Research Loma Linda University | School of Behavioral Health and Research, Behavioral Health Institute, Griggs Hall, 224, 11065 Campus Street, Loma Linda, CA 92350 USA

**Keywords:** Cannabis, Alcohol, Risk behaviors, Frequency of use

## Abstract

**Background:**

While the link between frequent cannabis use and alcohol use disorders is well documented, it is not clear whether alcohol drinkers who use cannabis less frequently are also vulnerable to alcohol use disorders. We estimate the association of frequency of past 12-months cannabis use with alcohol-associated adverse effects variables in the same time frame: alcohol dependence, heavy drinking, driving under alcohol influence, alcohol-related interpersonal problems, use after interpersonal problems, alcohol-related risky behaviors, and alcohol-related legal problems.

**Methods:**

We analyzed data from U.S. individuals aged 12 to 25 years who participated in annual, cross-sectional U.S. National Surveys on Drug Use and Health from 2002 to 2014. Logistic regression models were used to examine the association of cannabis use with six alcohol-associated adverse effects variables. Frequency of cannabis use served as the primary independent variable, and were divided into four categories: frequent use (21–30 days per month), less frequent use (1–20 days per month), no use over the past 12 months, and no lifetime cannabis use. Alcohol dependence and six alcohol-associated adverse effects variables served as our primary outcomes.

**Results:**

The study included 465,090 respondents aged 12 to 25 years, among all past-year cannabis users, (47.5%) were less frequent (1–20 days/month) users. Less frequent cannabis use was highest among male, 15–25-year-olds, and non-Hispanic white 11.8, 84 and 10.6%, respectively. In adjusted models, past-year less frequent cannabis use (1–20 days/month) was significantly associated with past-year alcohol dependence (adjusted odds ratio aOR 5.57, 95% confidence interval (CI) 5.5–6.4); heavy drinking in the past-year (aOR 3.41, 95% CI 3.2–3.5); alcohol-related interpersonal problems in the past-year (aOR 7.33, 95% CI 7.0–7.5); use after interpersonal problems (aOR 5.17, 95% CI 4.8–5.5); alcohol-related risky behaviors (aOR 7.29, 95% CI 7.0–7.5), and, driving under influence of alcohol (aOR 7.19, 95% CI 6.9–7.4). No cannabis use past-year were more likely to report alcohol dependence (aOR 2.81, 95% CI 2.6–3) compared with no lifetime cannabis use.

**Conclusion:**

These findings indicated that within the general population, not only frequent cannabis user (21–30 days per month) but even less frequent cannabis use (1–20 days/month) was significantly associated with past-year alcohol dependence and alcohol-associated adverse effects than no lifetime cannabis use. These adverse alcohol-related outcomes associated with less frequent cannabis use, should be taken under careful consideration in alcohol use disorder treatment setting and policy planning.

## Background

Cannabis use is widespread among adolescents and young adults. In 2014, 2.5 million individuals aged 12 of older reported cannabis use in the last month with 7000 new users every day, where 53% of this population was between 12 to 25 years old (Azofeifa et al. 2016; Burns et al. 2013). The prevalence of cannabis use among adolescents is at its highest rate in 30 years (Azofeifa et al. 2016), a trend largely attributed to the decreased risk perception of cannabis use from 50.4 to 33.3% in the last decade, (Miech et al. 2017; Mauro et al. 2018; Compton et al. 2016). Cannabis use is prospectively associated with both heavy drinking and with the development and maintenance of alcohol use disorders (AUDs) (Blanco et al. 2016; Metrik et al. 2012; Subbaraman and Kerr 2015). Cannabis dependence doubles the risk for long-term continual alcohol-related problems (Copeland et al. 2012), and cannabis- dependence among alcohol users are three times more likely to develop alcohol dependence than non-cannabis users (Metrik et al. 2012).

Cannabis is the most common substance used by adolescents and young adults who drink alcohol (Subbaraman and Kerr 2015), with almost one in four of individuals with past-year alcohol use disorder (AUD) reporting past-year cannabis use (Subbaraman et al. 2017). Cannabis use, particularly daily use, is associated with a variety of adverse correlates and consequences (Blanco et al. 2016; Volkow et al. 2014). These include substance use disorder, driving impairments, and motor vehicle crash injuries (Blanco et al. 2016; Hartman et al. 2015b; Metrik et al. 2012). Cannabis use has long been linked to heavy drinking and the development of alcohol use disorders (AUDs), both cross-sectionally and prospectively (Blanco et al. 2016; Subbaraman and Kerr 2015). While the link between daily cannabis use and AUDs is well documented, it is not clear whether this relationship is consistent with less frequent cannabis use, or whether drinkers who use cannabis less frequently are more likely to have AUD than those who do not.

There is a growing body of evidence linking cannabis use in youths and young adults with alcohol-associated adverse effects. However, most previously conducted studies focused on frequent cannabis users, which presumably led to higher levels of drinking by resulting in greater tendency to drink (Roche et al. 2019; and Lipari and Van Horn 2017). Several longitudinal studies have similarly concluded that frequent cannabis use is associated with an increased risk of developing alcohol dependency and other substance-related disorders (Buu et al. 2015; and Gunn et al. 2018). Gunn’s timeline follows back study found that on days on which cannabis was used, participants reported a higher number of drinks consumed compared to days when cannabis was not used (Gunn et al. 2018). Thus, there is an association between cannabis use and alcohol use, although more is known about alcohol use among frequent cannabis users. It is imperative to differentiate between frequent and less frequent cannabis use because harmful effects have different developmental correlates and consequences across different patterns of cannabis use (Fergusson et al. 2015). Building on previous research, we describe associations between less frequent cannabis use with alcohol dependence and with alcohol-related outcomes among youths and young adults (aged 12–25).

## Methods

Data were obtained from the annual 2002–2014 datasets of the U.S. National Survey on Drug Use and Health (NSDUH). The weighted interview response rate for these 13 years was 71.20% (Substance Abuse and Mental Health Data Archive (SAMHDA) 2014). Face-to-face interviews in U.S. residential households were used to collect the data, while the Research Triangle Institute in North Carolina prepared the data for public use files (PUFs) (Substance Abuse and Mental Health Data Archive (SAMHDA) 2014). The surveys used audio computer-assisted self-interviews (ACASI) to explore respondents’ substance use and other risk behaviors. Respondents were selected using a multistage area probability sample of the 50 states and the District of Columbia, resulting in a total annual sample of approximately 70,000 individuals, collected from each state in proportion to the population of the respective states. Methods for all years are described elsewhere (Substance Abuse and Mental Health Data Archive (SAMHDA) 2014). Conducted by the federal government since 1971, The National Survey on Drug Use and Health is the primary source of statistical information on the use of illegal drugs by the U.S. population. The reliability measure and consistency of respondents have been tested several times to ensure the accuracy of estimates (Substance Abuse and Mental Health Data Archive (SAMHDA) 2014).

The total cohort sample for these 13 years was 465,090 for participants aged 12 to 25 years old. A multistage sampling design was used, oversampling youths’ participants (aged 12–17 years) and young adults (aged 18–25 years) to allow increased precision in those age groups. Age was categorized into four groups: total 12–17 years, 18–25 years, and ≥ 26 years. Additional information about sample design can be found elsewhere (Center for Behavioral Health Statistics and Quality 2014).

### Alcohol-associated adverse effects

Six variables describe alcohol-related outcomes served as the primary outcomes: past-year alcohol dependence, heavy drinking in the past-year, alcohol-related interpersonal problems in the past-year, continued use after interpersonal problems, alcohol-related risky behaviors, and driving under the influence of alcohol. NSDUH collects information on past month and year alcohol use, binge alcohol use, and heavy alcohol use. For men, binge alcohol use is defined in NSDUH as drinking five or more drinks on the same occasion on at least 1 day in the past 30 days. For women, binge drinking is defined as drinking four or more drinks on the same occasion on at least 1 day in the past 30 days. Heavy alcohol use is defined as binge drinking on 5 or more days in the past 30 days (Center for Behavioral Health Statistics and Quality 2018).

Meeting criteria for dependence or abuse of alcohol or illicit drugs in the past year (“dependence or abuse”), a series of questions were asked by NSDUH. These questions are designed to measure symptoms of dependence and abuse based on criteria specified in the fourth edition of the Diagnostic and Statistical Manual of Mental Disorders (DSM-IV) (American Psychiatric Association 1994; Ahrnsbrak et al. 2017), including withdrawal, tolerance, use in dangerous situations, trouble with the law and interference in major obligations at work, school, or home during the past year. NSDUH also allows for estimating of alcohol use disorder and illicit drug use disorder.

### Predictor and covariates

Our primary exposure of interest was self-reported frequency of cannabis use over the past-year (1–20 days/month). Participants were asked if they ever used cannabis or hashish in their life. Those who reported “yes” were asked further follow-up questions about their frequency of use in the past month and year. To focus our analysis on less frequent use of cannabis and to assure the groups are mutually exclusive, cannabis users were divided into four categories: no lifetime cannabis use, no use over the past 12 months, frequent use (21–30 days per month), and less frequent use (1–20 days per month**)**. Unlike prior studies, in describing cannabis use behavior, we used “less frequent use 1-20 days per month” and “more frequent use 21-30 days per month” rather than “daily or near-daily use”, which more frequent users included a range of less than daily use (Azofeifa et al. 2016; Burns et al. 2013). Guided by previous studies (Azofeifa et al. 2016; Blanco et al. 2016; and Compton et al. 2016), demographics (age, race/ethnicity, and gender), tobacco smoking in past year, and using illicit drug rather than cannabis, were associated with cannabis use and alcohol use, and were classified as potential confounders. Gender was categorized into males and females. Race/ethnicity was categorized into four groups: non-Hispanic white, non-Hispanic black or African American, Hispanic or Latino, and others.

### Statistical analysis

Analyses were conducted using SAS 9.4. Survey-provided sampling weights were divided by the number of survey years and used to derive nationally representative estimates accounting for the NSUDH complex survey design. To examine the prevalence of frequent cannabis use, less frequent cannabis use, those who did not use cannabis in the past -year, and no lifetime cannabis use by age, gender, ethnicity, past-year alcohol dependence, heavy drinking in the past-year, alcohol-related interpersonal problems in the past-year, continued use after interpersonal problems, alcohol-related risky behaviors, and driving under the influence of alcohol, we calculated weighted proportions using survey-provided sampling weights (Peters and Chien 2018).

Multivariate binary and multinomial logistic regression models were used to examine the association of frequency of cannabis use (independent variable) with six alcohol-associated adverse effects variables (dependent variables). We first obtained univariate unadjusted estimates of the observed associations and then performed multivariate logistic regression models adjusted for potential confounders. All logistic regression models were adjusted for age, gender, race/ethnicity, tobacco smoking and using illicit drugs rather than cannabis (Hasin et al. 2017; Hasin 2018; and Coughlin et al. 2019). To estimate the subpopulation age domain of interest (12–25) in addition to the entire study population (age group 26 to 115) domain statement in SAS was used (Gossett et al. 2002). Domain analysis allowed us to calculate the computation of statistics for domain age group 12 to 25 (subpopulations) in addition to the computation of statistics for the entire study population (age group 26 to 115). Multicollinearity effect of examined explanatory variables was ruled out using variance inflation factors.

## Results

### Past-year cannabis use by demographic characteristics of respondents

Model-based prevalence of past-year frequency of cannabis use by demographic characteristics of survey respondents are shown in Table 1. The study sample of individuals aged 12 to 25 years old included 465,090 respondents in the 2002–14 surveys. Less frequent cannabis use was highest among male, 15–25-year-olds, and non-Hispanic white 11.8, 84 and 10.6%, respectively. Nearly, half of past-year cannabis users reported less frequent (1–20 days/month) cannabis use 47.5%. The overall prevalence of individuals reported less frequent past-year cannabis use (1–20 days per month) was 10.5%, whereas frequent cannabis users (21–30 days/month), no past-year cannabis use, and no lifetime cannabis use were 11.6, 13.8, and 64%, respectively.

**Table 1:** Past-year cannabis use by demographic characteristics of respondents ^a^

Characteristics of cannabis Use	(1–20 days per month)	21–30 days per month)	No past 12 months use	No lifetime use	***P-***Value^**b**^
	***n*** = 48,661 (10.5)	***n*** = 53,805 (11.6)	***n*** = 64,425 (13.8)	***n*** = 298,199 (64)	
	No.(Row.%)	No.(Row.%)	No.(Row.%)	No.(Row.%)	
**Age**					<.001
12	204 (1)	219 (0.7)	159 (0.4)	35,331 (98.4)	
13	742 (2)	786 (2.1)	425 (1.1)	36,193 (94.9)	
14	1843 (4.8)	1829 (4.8)	890 (2.3)	33,874 (88.1)	
15	3406 (8.7)	3383 (8.6)	1620 (4.1)	30,913 (78.6)	
16	4870 (12.3)	4739 (12)	2550 (6.4)	27,451 (69.3)	
17	5659 (14.6)	5847 (15.1)	3411 (8.8)	23,923 (61.6)	
18	4941 (15.2)	5671 (17.4)	3708 (11.4)	18,278 (56.1)	
19	4800 (16.2)	5248 (17.7)	4432 (14.9)	15,195 (51.2)	
20	4476 (15.5)	5175 (10.7)	5667 (19.6)	13,623 (47.1)	
21	4297 (14.7)	4958 (17.9)	6951 (23.8)	13,020 (44.6)	
22 or 23	7339 (12.7)	8730 (17)	16,327 (28.3)	25,334 (43.9)	
24 or 25	6084 (10.7)	7220 (15.1)	18,285 (32.3)	25,064 (44.3)	
**Gender**					<.001
Male	26,961 (11.8)	28,988 (12.6)	29,459 (12.9)	143,841 (62.7)	
Female	21,700 (9.2)	24,817 (10.5)	34,966 (14.8)	154,358 (65.5)	
**Race/Ethnicity**					<.001
Non-Hispanic White	29,748 (10.6)	35,715 (12.3)	40,988 (14.6)	173,645 (62)	
Non-Hispanic Black/African American	7138 (11.4)	6282 (10)	7420 (13.2)	42,022 (66.9)	
Hispanic	7450 (9.3)	7229 (9)	10,604 (13.2)	54,830 (68.4)	
Other Race	4325 (10.3)	4579 (10.9)	5413 (12.9)	27,702 (65.9)	

*Abbreviations*: *n* is actual sample size, *No* number of participants**,** *Row* Row percentage presented

^a^ Data are from the U.S. National Survey on Drug Use and Health 2002–2014 age 12 to 25 years old

^b^ *P*-values are from binary analysis using a χ2 Chi-square test for all variables (categorical)

### Past-year alcohol-associated adverse effects among past-year cannabis users

Model-based prevalence of past-year alcohol-associated adverse effects among cannabis users are shown in Table 2. Individuals reported alcohol dependence, alcohol related interpersonal problems, alcohol interpersonal problems, and alcohol-related legal problems were higher among less frequent cannabis users compared to cannabis frequent users, 32.9, 30.8, 33.2, and 36.4%, respectively. One in four individuals reported alcohol-related legal problems were less frequent cannabis users 25.6%. One in three of individuals reported past-year alcohol dependence were less frequent cannabis users 33%.

**Table 2:** Prevalence of past-year alcohol-associated adverse effects among past-year cannabis users ^a^

Alcohol related outcomes	(1–20 days per month)	(21–30 days per month)	No past 12 months use	No lifetime use	***P-***Value ^**b**^
	No. (Row.%)	No. (Row.%)	No. (Row.%)	No. (Row.%)	
	***n*** = 48,661 (10.5)	***n*** = 53,805 (11.6)	***n*** = 64,425 (13.8)	***n*** = 298,199 (64)	
**Alcohol dependence**					<.001
Yes	6679 (32.9)	63,139 (31.1)	4114 (20.3)	3203 (15.8)	
No	41,982 (9.4)	47,492 (10.7)	60,311 (13.6)	294,996 (66.3)	
**Heavy drinking**					<.001
Heavy drinking ^c^	23,395 (25.4)	24,422 (26.5)	22,769 (24.7)	21,599 (23.4)	
Binge drink ^d^	21,747 (13.3)	25,649 (15.7)	31,536 (19.8)	84,712 (51.8)	
Never drink	3519 (1.7)	3734 (1.8)	10,120 (4.8)	191,888 (91.7)	
**Driving under the influence of alcohol**					<.001
Yes	14,176 (25.6)	16,630 (30)	13,785 (24.9)	10,889 (19.6)	
No	30,665 (15.6)	33,178 (16.9)	40,230 (20.4)	92,724 (47.1)	
**Alcohol-related interpersonal problems**					<.001
Yes	5816 (30.8)	5645 (29.9)	3833 (20.3)	3586 (19)	
No	35,307 (19.6)	37,414 (20.7)	42,793 (23.7)	64,960 (36)	
**Continued use after interpersonal problems**					<.001
Yes	3798 (33.2)	3737 (32.7)	2240 (19.6)	1664 (14.6)	
No	2012 (27.1)	1902 (25.7)	1590 (21.1)	1910 (25.8)	
**Alcohol-related risky behaviors**					<.001
Yes	9975 (30.2)	10,539 (31.9)	6875 (20.8)	5608 (17)	
No	31,125 (18.7)	32,506 (19.6)	39,737 (23.9)	62,898 (37.8)	
**Alcohol-related legal problems**					<.001
Yes	2436 (36.4)	2142 (32)	1186 (17.7)	936 (13.4)	
No	38,691 (20.1)	40,913 (21.2)	45,437 (23.6)	67,597 (35.1)	

^a^ Data are from the U.S. National Survey on Drug Use and Health 2002–2014 age 12 to 25 years old

^b^ *P*-values refer to comparisons between two groups and were adjusted for age, race, gender, tobacco smoking in past year, and using illicit drug rather than cannabis and as covariates

^c^ Heavy alcohol drinking = as binge drinking 5 or more days in the past 30 days (20 > drinks a month)

^d^ Binge drink = drinking five or more drinks (for males) or four or more drinks (for females) on the same occasion

### Odds ratios of past-year cannabis use

Unadjusted and adjusted logistic regression findings are presented in Table 3. The patterns of findings in the unadjusted and adjusted models remained the same. Less frequent cannabis use was common among individuals reported alcohol-associated adverse effects. Unadjusted findings suggested that frequency of cannabis use was significantly associated with all alcohol-associated adverse effects. In the fully adjusted multivariable logistic regression models, less frequent cannabis use was significantly associated with past-year alcohol dependence (aOR 5.57, 95% CI 5.5–6.4); heavy drinking in the past-year (aOR 3.41, 95% CI 3.2–3.5); alcohol-related interpersonal problems in the past-year (aOR 7.33, 95% CI 7.0–7.5); continuing to drink after interpersonal problems (aOR 5.17, 95% CI 4.8–5.5); alcohol-related risky behaviors in the past-year (aOR 7.29, 95% CI 7.0–7.5), and, driving under the influence of alcohol (aOR 7.19, 95% CI 6.9–7.4) compared with no lifetime cannabis use. Individuals reported less frequent cannabis use had a consistently slightly greater likelihood of reporting alcohol-associated adverse effects than individuals reported frequent cannabis use except for driving under the influence of alcohol. Individuals reported no cannabis use past-year were more likely to report alcohol dependence (aOR 2.81, 95% CI 2.6–3) compared to no lifetime cannabis use.

**Table 3:** Multiple logistic regressions estimating association of Cannabis Use with alcohol-associated adverse effects (Unadjusted/Adjusted) ^a^

	(1–20 time per month) Cannabis Use ^b^		(21–30 times per month) Cannabis Use ^c^		No past 12 months use		No lifetime use	
	OR (95%Cl)		OR (95%Cl)		OR (95%Cl)		OR (95%Cl)	
	Unadjusted	Adjusted	Unadjusted	Adjusted	Unadjusted	Adjusted	Unadjusted	Adjusted
Alcohol dependence past year (Yes Vs. No)	**13.95 (13.1–14.8)**	**5.57 (5.5–6.4)**	11.71 (11.1–12.3)	5.06 (4.7–5.3)	6.14 (5.7–6.5)	2.81 (2.6–3.0)	Ref	Ref
Heavy drinking (Heavy drinking Vs. binge drink)	**15.61 (15.1–16.1)**	**3.41 (3.2–3.5)**	14.28 (13.9–14.6)	2.86 (2.6–2.9)	8.62 (8.3–8.8)	1.76 (1.7–1.9)	Ref	Ref
Driving under the influence of alcohol (Yes Vs. No)	16.11 (15.5–16.6)	7.19 (7.4–9.6)	**17.05 (16.6–17.5)**	**7.29 (7.1–7.5)**	9.31 (9.0–9.6)	3.06 (2.7–3.1)	Ref	Ref
Alcohol-related interpersonal problems past year (Yes Vs. No)	**16.91 (16.3–17.4)**	**7.33 (7.0–7.5)**	13.41 (13.0–13.8)	5.51 (5.3–5.6)	8.21 (8.0–8.4)	2.76 (2.6–2.8)	Ref	Ref
Continued use after interpersonal problems past year (Yes Vs. No)	**10.58 (10.0–11.2)**	**5.17 (4.8–5.5)**	9.16 (8.7–9.6)	4.60 (4.3–4.9)	5.00 (4.6–5.3)	2.69 (2.4–2.9)	Ref	Ref
Alcohol-related risky behaviors past 12 months (Yes Vs. No)	**16.67 (16.1–17.1)**	**7.29 (7.0–7.5)**	14.15 (13.7–14.5)	5.82 (5.6–5.9)	8.07 (7.8–8.2)	2.74 (2.6–2.8)	Ref	Ref
Alcohol-related legal problems past 12 months (Yes Vs. No)	18.44 (17.8–19.1)	**8.45 (8.1–8.7)**	13.68 (13.2–14.1)	5.88 (5.6–6.1)	8.33 (8.3–8.5)	2.75 (2.6–2.8)	Ref	Ref

Bolded estimates indicate differences at the *p* < 0.001 level of significance with the highest odds ratio

Adjusted for age, race, gender, tobacco smoking in past year, and using illicit drug rather than cannabis and as covariates

*aOR* Adjusted odds ratio, *CI* confidence Interval, *Ref* Reference

^a^ Data are from the U.S. National Survey on Drug Use and Health 2002–2014 age 12 to 25 years old

^b^ (1–20 days/month): those who reported 1–20 days a month using cannabis

^c^ (21–30 days/month): those who reported 21–30 days a month using cannabis

## Discussion

The study examined the association between frequency of cannabis use and reported alcohol-associated adverse effects across 13 years of nationally representative data in the United States. Three key findings emerged from this analysis. First, for all findings, frequency of cannabis use was significantly associated with past-year alcohol dependence and alcohol-associated adverse effects. Second, this study indicates that not only frequent cannabis users but even those who use cannabis less frequent are more likely to report alcohol-associated adverse effects compared to those who report no lifetime cannabis use. Third key finding, even those who reported not using cannabis in the past-year, but did use cannabis in the past are also more likely to report past-year alcohol dependence and alcohol-associated adverse effects compared to no lifetime cannabis use. These associations are particularly salient among those who reported using cannabis less frequently (1–20 days/month). In fact, this study found that about 33% of those who reported past-year alcohol dependence were less frequent (1–20 days/month) cannabis users.

While previous studies concluded that among alcohol dependent individuals, using cannabis is associated with alcohol consumption (Subbaraman et al. 2017; and Mikuriya 2004), this study demonstrates that regardless of the frequency of cannabis use, individuals who used cannabis in the past-year are more likely to report past-year heavy alcohol drinking, alcohol dependence and other alcohol-associated adverse effects, compared to those who never used cannabis but did use alcohol in the past-year. Moreover, alcohol dependence rate was more than double among cannabis users compared to those who never used cannabis. Our findings extend previous findings that have focused on older population or a restricted pattern of cannabis use. In Blanco’s study, no quantification of cannabis use level was examined (Blanco et al. 2016). The study did not identify which level of cannabis consumption was more likely to report alcohol disorders. Other research has suggested that a higher frequency of cannabis use is significantly associated with an increased probability of reporting alcohol use disorder (Blazer and Wu 2009). This study, however, examined older adults (aged 50 or older) (Lloyd and Striley 2018), who have different characteristics than younger population. The current study found that, compared to those who reported no lifetime cannabis use, adolescents and youths with less frequent cannabis use (1–20 days/month) are more likely to report alcohol dependence and alcohol-associated adverse effects.

The finding that cannabis use was associated heavy drinking and alcohol-associated adverse effects may be explained by increased impulsivity, risk taking, and reduced inhibitory control from using cannabis (Metrik et al. 2012). Impulsivity is associated with increased risk for addiction disorders, engagement in risk behaviors, and alcohol related disorder (Ansell et al. 2015; Metrik et al. 2012). Alternatively, a simultaneous use of alcohol and cannabis produces significantly higher blood concentrations of cannabis’s main psychoactive chemical components, tetrahydrocannabinol (THC) that affect brain areas related to pleasure sensation (Hartman et al. 2015a, 2015b). While less frequent cannabis users consume lower amount of THC (Fischer et al. 2017), some individuals might use cannabis purposefully and simultaneously with alcohol in order to enhance cannabis’s pleasurable effects that resulted from THC (Subbaraman and Kerr 2015).

The effects of a drug (legal or illegal) on individual risk behaviors are determined not only by its pharmacologic properties but also by its availability and social acceptability (Volkow et al. 2014). As more states legalize or decriminalize cannabis use, its use becomes more accepted across the nation, and it is expected that prevalence of cannabis use will continue rise among adolescents and young adults (Cerda et al. 2020; and Compton et al. 2016). Therefore, our findings suggest caution in the implementation of policies related to legalization of cannabis for recreational use, as it may lead to greater availability and acceptance of cannabis (Blanco et al. 2016), reduced perception of risk of use (Miech et al. 2017), higher number of less frequent cannabis users (Mauro et al. 2018), and increased risk of alcohol related disorder (2018; Lipari et al. 2017).

Three design factors strengthen our study: the large sample size in each of the 13 waves from 2002 to 2014, the national population-based sampling strategy with a high response rate, and the rigorous protocols used to assess detailed information with respect to cannabis use. The consistency of findings across various frequency levels of cannabis use strengthens inferences regarding the association of cannabis use patterns with alcohol-associated adverse effects. There are, however, systematic differences in the amount of the active compound consumed per day across different groups of users (Casajuana et al. 2016; Kilmer et al. 2013). Typically, those who use cannabis more days per month also tend to use more grams per day, which is a variable that we did not control for (Casajuana et al. 2016; Kilmer et al. 2013).

These findings should be interpreted with some caution. First, the cross-sectional nature of our data precludes drawing causal inference related to the associations we have reported. Second, substance use behaviors were determined from respondents’ self-reports, which are subject to a variety of biases associated with memory errors and recall. Third, the survey items did not differentiate between cannabis product types such as sativa, indica, or hybrid with varying psychoactive THC concentrations. In addition, the number times of cannabis use per day were not included in the surveys. These distinctions can be important because a different type of cannabis might result in a different outcome, although addressing this issue goes beyond any monitoring study (Ahrnsbrak et al. 2017).

## Conclusion

Our study indicates that past-year less frequent cannabis use (1–20 days/month) was significantly associated with alcohol dependence and alcohol-associated adverse effects. In a nationally representative sample of adolescents and youth in the U.S., the current study demonstrates that individuals with less frequent cannabis use were more likely to report past-year alcohol dependence and alcohol-associated adverse effects, compared to individuals with no lifetime cannabis use. Additionally, individuals reported no cannabis use past-year but did use in the past, were also more likely to report alcohol dependence compared to no lifetime cannabis use. These adverse alcohol-related outcomes associated with less frequent cannabis use, should be taken under careful consideration in clinical care and policy planning for future cannabis legalization. Clinicians who treat patients with substance use disorders, should consider screening their patients for cannabis use patterns to determine the optimal treatment options. Future studies should consider a more comprehensive analysis of cannabis use patterns to establish the impact of different patterns of cannabis use on alcohol-associated adverse effects.

## Data Availability

The data that support the findings of this study are openly available in [National Survey on Drug Use and Health] at [https://www.datafiles.samhsa.gov/study-dataset/national-survey-drug-use-and-health-2002-2014-nsduh-2002-2014-ds0001-nid16960].
